# Association of the combined atherosclerosis index of plasma and frailty index with arthritis in middle-aged and older adults: a cohort study

**DOI:** 10.1038/s41598-026-58737-y

**Published:** 2026-06-26

**Authors:** Bo Chen, Xiangyi Li

**Affiliations:** 1 Department of Orthopaedics, The Second People’s Hospital of Changzhi City, Shanxi, China; 2 Graduate School, Changzhi Medical College, Shanxi, China

**Keywords:** Atherogenic index of plasma, Frailty index, Arthritis, CHARLS, Cohort study, Cardiology, Diseases, Health care, Medical research, Rheumatology, Risk factors

## Abstract

The Atherogenic Index of Plasma (AIP) is a marker of atherosclerosis, and the Frailty Index (FI) reflects physiological decline. The combined effect of AIP and FI remains insufficiently studied. This study aimed to investigate the association between a combined AIP-FI measure and the risk of arthritis. This study utilized data from the China Health and Retirement Longitudinal Study (CHARLS) database between 2011 and 2020. A total of 4437 participants aged ≥ 45 years without arthritis at baseline were included. Cox proportional hazards regression models and restricted cubic spline (RCS) models were employed to explore the relationship between the AIP-FI and the risk of incident arthritis. Over a 10-year follow-up period, a total of 1470 participants developed arthritis, with an incidence rate of 33.13%. Cox regression analysis revealed that each 1-unit increase in AIP-FI was associated with a 3% increase in arthritis risk (HR = 1.03; 95% CI: 1.02 to 1.04). Participants in the highest tertile (T3) of AIP-FI had a 55% higher risk compared to those in the lowest tertile (T1) (HR = 1.55; 95% CI: 1.35 to 1.78). The RCS curve demonstrated a significant nonlinear positive association between AIP-FI and arthritis risk (P for overall < 0.001, P for nonlinear < 0.001). This study indicates that a higher AIP-FI is significantly associated with an increased risk of arthritis. By integrating metabolic and frailty data, the AIP-FI was associated with arthritis risk and may help identify older Chinese adults at higher risk. However, these findings require further validation in independent cohorts before any clinical consideration.

**Supplementary Information:**

The online version contains supplementary material available at https://doi.org/10.1038/s41598-026-58737-y.

## Introduction

With population aging and the increasing prevalence of obesity, the prevalence of osteoarthritis (OA) is rising annually^1,2^. Data from 2020 indicate that the global burden of OA continues to escalate, currently affecting approximately 7.6% of the global population, with projections suggesting a 60%–100% increase in prevalent cases by 2050^3^. Furthermore, OA-related joint pain, particularly from knee OA^4,5^, leads to functional impairment^4,6^, loss of independence in daily life^4,6^, reduced sleep quality, fatigue^4,7,8^, depressed mood^4,9,10^, and is the primary indication for joint replacement surgery^4,9,11–14^. Therefore, arthritis represents a prevalent condition associated with considerable societal and individual costs, and the identification of accessible risk assessment indicators is essential for its timely detection and management.

The atherogenic index of plasma (AIP), defined as the logarithmic ratio of triglycerides to high-density lipoprotein cholesterol, is a sensitive marker of the plasma lipoprotein profile^15^. Ge et al.^16^ reported that elevated AIP was associated with increased arthritis prevalence, with the highest quartile showing a 45% higher risk than the lowest after multivariable adjustment (OR = 1.45, 95% CI: 1.20–1.74). Additionally, Xu et al.^17^, using NHANES data, found a significant nonlinear positive correlation between AIP and multi-site bone mineral density, particularly in females. The frailty index (FI), which quantifies cumulative deficits across physical, medical, cognitive, and psychological domains, is a robust indicator of biological aging^18^. Huang et al.^19^ identified an association between FI and osteoarthritis (OR = 1.07, 95% CI: 1.05–1.08), with Mendelian randomization further supporting causality (IVW OR = 1.69, 95% CI: 1.01–2.83). From a mechanistic perspective, oxidative stress and inflammatory responses constitute a shared pathological basis linking metabolic syndrome and frailty syndrome^20^. Dyslipidemia reflected by elevated AIP may promote oxidative stress and inflammatory reactions in joint tissues, thereby inducing chondrocyte apoptosis and cartilage degradation^21^. Frailty status captured by FI is associated with elevated inflammatory parameters, particularly CRP and IL-6^22^, which are closely linked to arthritis progression. This suggests that AIP and FI may jointly contribute to arthritis development through shared oxidative stress and inflammatory pathways. However, these studies examined AIP or FI separately; no study has explored their combined effect. Given that AIP reflects metabolic dysfunction while FI captures frailty status, their integration may offer more comprehensive risk assessment than either alone. This study therefore aimed to examine the association between combined AIP-FI and arthritis risk in a nationally representative sample of middle-aged and older adults.

## Methods

### Study design

This cohort study utilized data from the China Health and Retirement Longitudinal Study (CHARLS), a nationwide survey targeting middle-aged and elderly populations in China. The survey was initiated in 2011, recruiting approximately 17,700 participants from about 10,000 households across 150 county-level units and 450 village or resident communities. To date, five waves of follow-up surveys have been completed (in 2011, 2013, 2015, 2018, and 2020)^23^. The CHARLS study was approved by the Ethical Review Committee of Peking University, and all participants provided written informed consent prior to enrollment.

### Study population

**Fig. 1:**
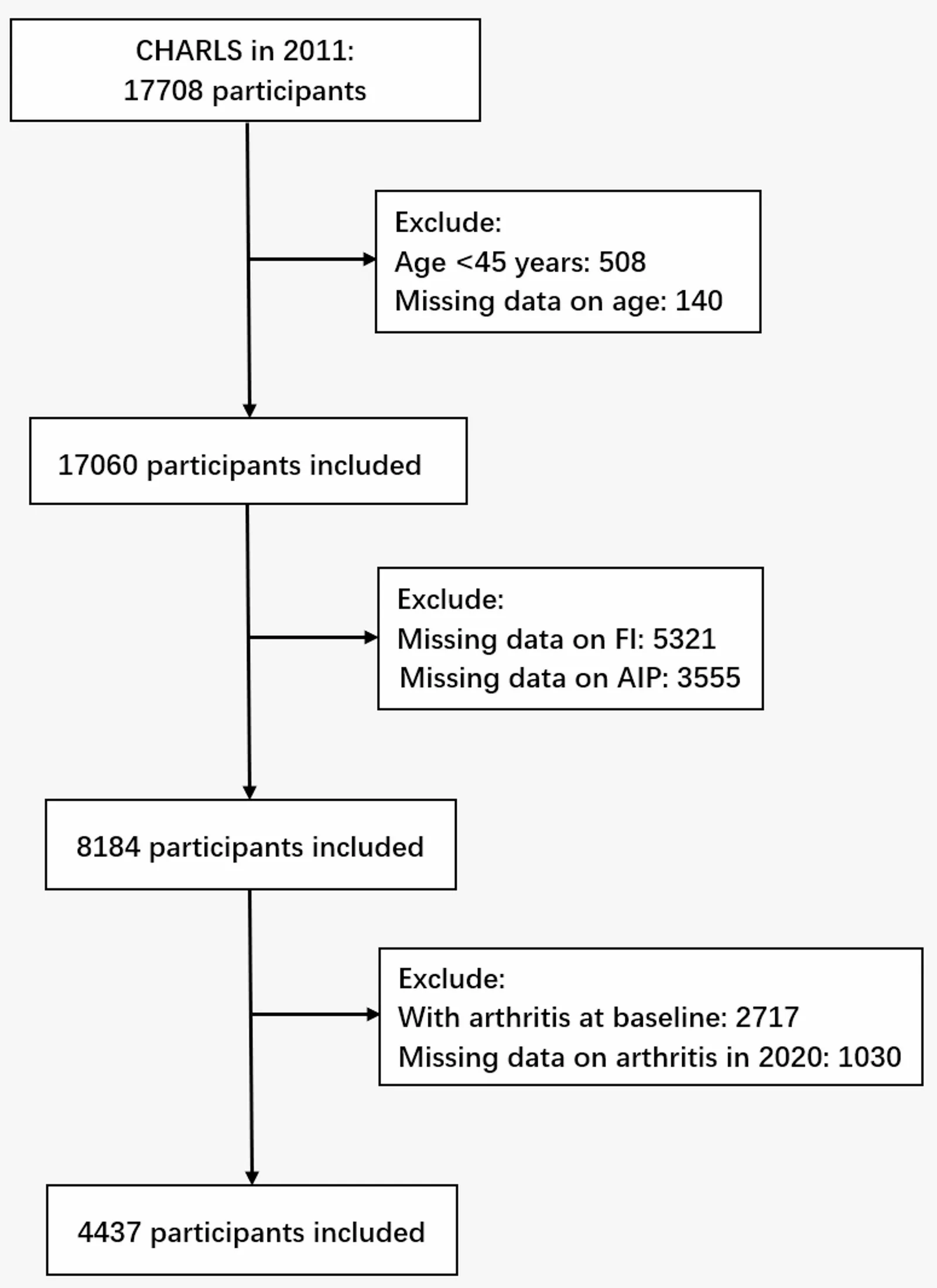
Participant screening process.

In this study, participants were excluded if they were younger than 45 years at baseline, had arthritis at baseline, could not be assessed for the AIP-FI, or lacked outcome data at the end of follow-up. A final sample of 4,437 eligible participants was obtained. The participant selection process is illustrated in Fig. 1.

### Data collection

Baseline information was collected by trained interviewers using standardized questionnaires. The collected data included: age, marital status, residence, sex, educational level, BMI, smoking status, alcohol consumption, diabetes, heart disease, stroke, dyslipidemia, hypertension, triglycerides (TG), high-density lipoprotein cholesterol (HDL-C), and arthritis.

### Calculation of AIP-FI

The Frailty Index (FI) in this study was calculated using the method reported in prior literature, incorporating 32 health deficits across somatic function, cognitive-psychological status, and chronic disease conditions. Except for cognitive scores (ranging from 0 to 1), each item was scored as 0 (no deficit) or 1 (deficit present). The individual FI was calculated using the formula: FI = (sum of deficit scores / 32) × 100, with higher values indicating greater frailty^24^. The specific items are provided in Supplementary Table 1. The Atherogenic Index of Plasma (AIP) was calculated using the formula: AIP = log₁₀(TG / HDL-C). A composite indicator AIP-FI was constructed using a multiplicative model: AIP-FI = AIP × FI. This approach has been applied in cardiovascular risk studies within the CHARLS cohort^18^. AIP reflects metabolic dysfunction, whereas FI captures physiological decline; representing distinct pathological dimensions, the multiplicative model was designed to capture the potential combined effect between the two.

### Covariates and definition of arthritis

The covariates included in this study were selected based on established risk factors for arthritis and variables associated with AIP or FI, covering demographic characteristics, lifestyle factors, and clinical metabolic indicators. Diabetes, heart disease, stroke, dyslipidemia, and hypertension were identified based on self-reported diagnosis by a physician. Incident arthritis was determined according to self-reported data in response to the question: “Have you been diagnosed with arthritis by a doctor?” An affirmative answer classified the participant as having arthritis. Participants who reported having arthritis in 2011 were excluded. Those diagnosed with arthritis thereafter until 2020 were defined as incident arthritis cases.

### Statistical analysis

Before imputation of baseline covariate data, the number and proportion of missing values for each covariate were as follows: dyslipidemia, 31 (0.70%); BMI, 488 (11.00%); and no missing values were observed for all remaining covariates. Given that the missing proportions for all covariates were low (< 15%), predictive mean matching (PMM) was used for imputation of continuous variables, whereas logistic regression was employed for categorical variables. Quantitative data are presented as mean ± standard deviation and were assessed using analysis of variance (ANOVA); categorical data are presented as frequency and percentage and were analyzed using the chi-square test.

AIP-FI was analyzed both as a continuous variable and as a categorical variable. The distribution characteristics of AIP, FI, and AIP-FI are presented in Supplementary Fig. 1: AIP approximated a symmetric distribution (mean 0.36, median 0.33), FI exhibited a right-skewed distribution (mean 9.85, median 7.92), and AIP-FI showed a markedly right-skewed distribution (mean 3.70, median 2.09, range − 13.43 to 67.15). Although continuous variable analysis can reflect overall trends, given the skewed distribution of AIP-FI and the presence of negative values (arising when HDL-C exceeds TG, resulting in a negative AIP), we further categorized it by tertiles to enhance clinical interpretability and reduce the influence of extreme values. For categorical analysis, the tertile cutoff values were 0.94 (P33) and 3.92 (P67), forming three groups: T1 (< 0.94), T2 (0.94–3.92), and T3 (> 3.92), with T1 serving as the reference group. In the association analysis, we used Cox proportional hazards regression models to assess hazard ratios (HR) and 95% confidence intervals (95% CI) for the relationship between AIP-FI and the incidence of arthritis. The proportional hazards assumption was assessed using Schoenfeld residuals. The global test showed no significant violation (χ² = 22.52, df = 22, *P* = 0.429), supporting the appropriateness of the Cox proportional hazards model. Three models were employed: Model 1 was unadjusted; Model 2 adjusted for age, marital status, residence, sex, and educational level; Model 3 further adjusted for diabetes, heart disease, stroke, dyslipidemia, BMI, hypertension, smoking, and alcohol consumption based on Model 2. This study also utilized restricted cubic splines (RCS) to examine the nonlinear relationship between AIP-FI and arthritis. Subgroup analyses were conducted to explore the correlation between AIP-FI and arthritis across different subgroups. Data management and statistical analyses were performed using the Storm Statistical Platform or Zstats software (www.zstats.net) and R version 4.4.0 (2024-04-24). A two-sided P-value < 0.05 was considered statistically significant.

## Results

### Baseline characteristics of the study population

This study included a total of 4,437 participants, categorized into three AIP-FI groups: T1 (*n* = 1,464, 33.00%), T2 (*n* = 1,509, 34.00%), and T3 (*n* = 1,464, 33.00%). Statistically significant differences (*P* < 0.05) were observed across the three groups for diabetes, heart disease, stroke, dyslipidemia, BMI, hypertension, education level, smoking status, alcohol consumption, sex, residence, arthritis, and age. In contrast, no significant difference was found for marital status (*P* > 0.05). From T1 to T3, there was a progressive increase in the proportions of individuals with diabetes, heart disease, stroke, hypertension, dyslipidemia, BMI ≥ 28 kg/m², and females. Conversely, the proportions of current smokers, alcohol consumers, and males gradually decreased. Details are presented in Table 1.

**Table 1:** Characteristics of the study population.

Variables	Total (*n* = 4437)	T1 (*n* = 1464)	T2 (*n* = 1509)	T3 (*n* = 1464)	Statistic	*P*
Diabetes					χ²=149.37	**< 0.001**
No	4186 (94.34)	1437 (98.16)	1455 (96.42)	1294 (88.39)		
Yes	251 (5.66)	27 (1.84)	54 (3.58)	170 (11.61)		
Heart disease					χ²=219.39	**< 0.001**
No	4060 (91.50)	1415 (96.65)	1434 (95.03)	1211 (82.72)		
Yes	377 (8.50)	49 (3.35)	75 (4.97)	253 (17.28)		
Stroke					χ²=68.47	**< 0.001**
No	4373 (98.56)	1459 (99.66)	1502 (99.54)	1412 (96.45)		
Yes	64 (1.44)	5 (0.34)	7 (0.46)	52 (3.55)		
Dyslipidemia					χ²=161.92	**< 0.001**
No	4025 (90.71)	1405 (95.97)	1405 (93.11)	1215 (82.99)		
Yes	412 (9.29)	59 (4.03)	104 (6.89)	249 (17.01)		
BMI					χ²=274.73	**< 0.001**
< 18.5	195 (4.39)	92 (6.28)	60 (3.98)	43 (2.94)		
18.5–23.9	2521 (56.82)	1019 (69.60)	850 (56.33)	652 (44.54)		
24.0-27.9	1288 (29.03)	297 (20.29)	450 (29.82)	541 (36.95)		
>=28	433 (9.76)	56 (3.83)	149 (9.87)	228 (15.57)		
Hypertension					χ²=445.79	**< 0.001**
No	3389 (76.38)	1318 (90.03)	1223 (81.05)	848 (57.92)		
Yes	1048 (23.62)	146 (9.97)	286 (18.95)	616 (42.08)		
Education					χ²=23.47	**< 0.001**
Below junior high school	2817 (63.49)	893 (61.00)	924 (61.23)	1000 (68.31)		
Junior high school	1014 (22.85)	356 (24.32)	363 (24.06)	295 (20.15)		
Senior high school	539 (12.15)	195 (13.32)	194 (12.86)	150 (10.25)		
University and above	67 (1.51)	20 (1.37)	28 (1.86)	19 (1.30)		
Smoking status					χ²=47.24	**< 0.001**
Never	2622 (59.09)	827 (56.49)	860 (56.99)	935 (63.87)		
Former	377 (8.50)	100 (6.83)	128 (8.48)	149 (10.18)		
Current	1438 (32.41)	537 (36.68)	521 (34.53)	380 (25.96)		
Drinking status					χ²=76.45	**< 0.001**
Never	2502 (56.39)	741 (50.61)	863 (57.19)	898 (61.34)		
Former	326 (7.35)	88 (6.01)	94 (6.23)	144 (9.84)		
Current	1609 (36.26)	635 (43.37)	552 (36.58)	422 (28.83)		
Gender					χ²=48.56	**< 0.001**
Women	2188 (49.31)	640 (43.72)	723 (47.91)	825 (56.35)		
Men	2249 (50.69)	824 (56.28)	786 (52.09)	639 (43.65)		
Residence					χ²=15.03	**< 0.001**
Urban	1690 (38.09)	499 (34.08)	599 (39.70)	592 (40.44)		
Rural	2747 (61.91)	965 (65.92)	910 (60.30)	872 (59.56)		
Marital status					χ²=10.80	0.095
Married	4092 (92.22)	1362 (93.03)	1398 (92.64)	1332 (90.98)		
Divorced	27 (0.61)	11 (0.75)	8 (0.53)	8 (0.55)		
Widowed	299 (6.74)	82 (5.60)	97 (6.43)	120 (8.20)		
Unmarried	19 (0.43)	9 (0.61)	6 (0.40)	4 (0.27)		
Arthritis status					χ²=48.37	**< 0.001**
No	2967 (66.87)	1051 (71.79)	1036 (68.65)	880 (60.11)		
Yes	1470 (33.13)	413 (28.21)	473 (31.35)	584 (39.89)		
Age					χ²=41.30	**< 0.001**
45–60	2818 (63.51)	1002 (68.44)	978 (64.81)	838 (57.24)		
>=60	1619 (36.49)	462 (31.56)	531 (35.19)	626 (42.76)		

χ²: Chi-square test; BMI, body mass index.

### Association between AIP-FI and arthritis risk

**Fig. 2:**
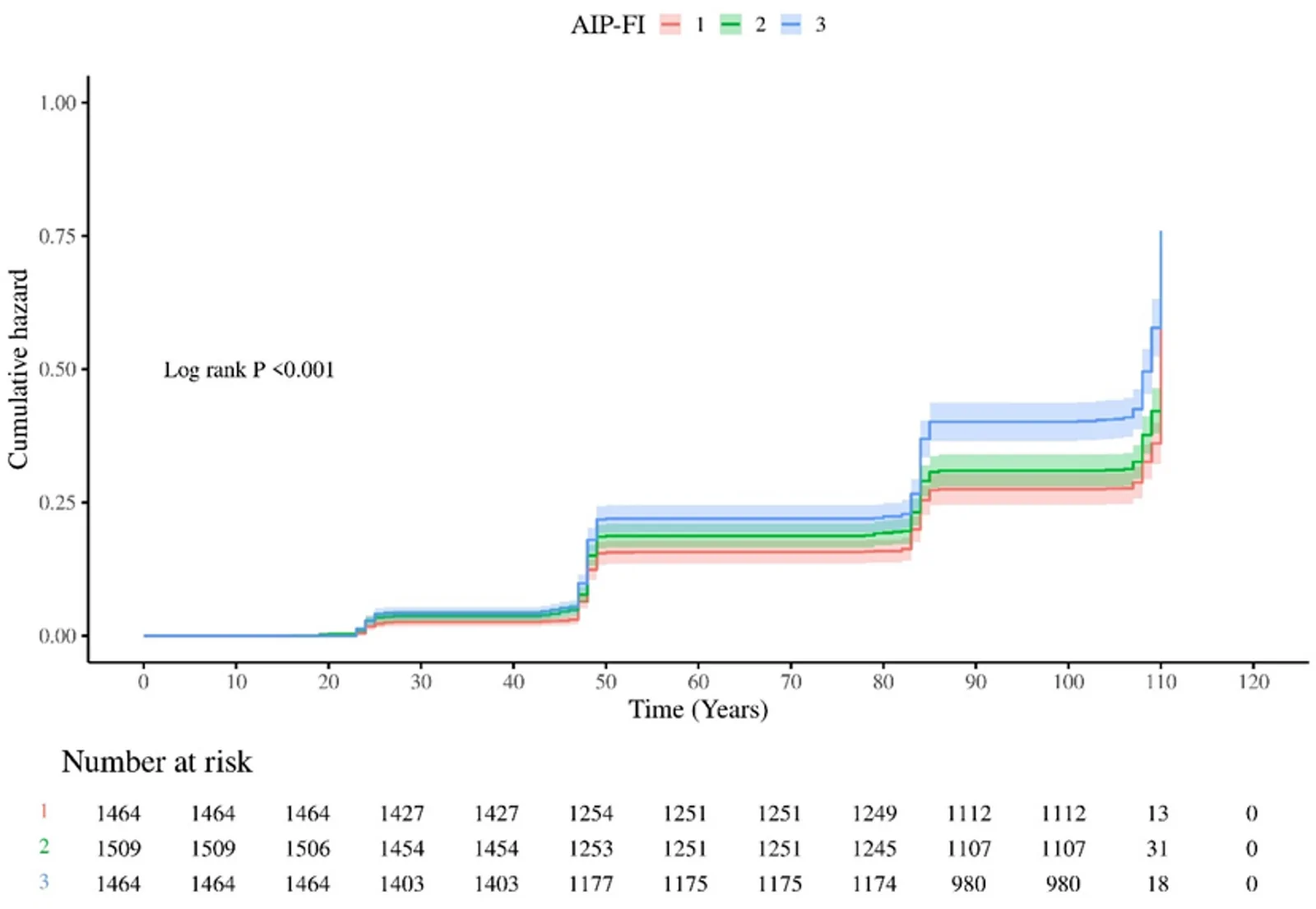
Kaplan-Meier curve for cumulative arthritis risk according to AIP-FI tertiles.

**Fig. 3:**
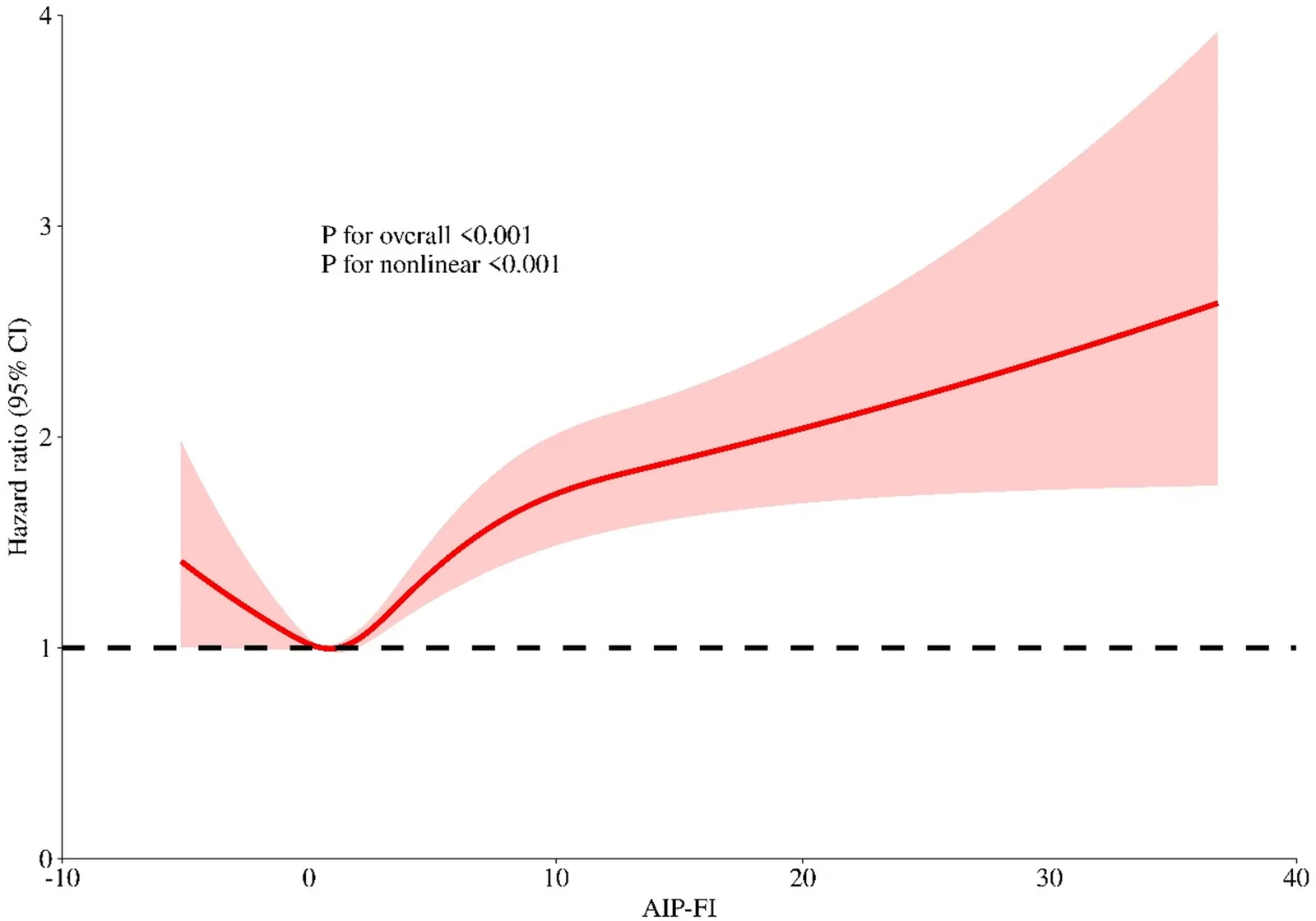
Restricted cubic spline curve for the association between AIP-FI and arthritis risk.

Table 1 presents the number and proportion of incident arthritis cases during the 10-year follow-up period. A total of 1,470 participants developed arthritis, corresponding to an incidence rate of 33.13%. The incidence rates across the tertile groups were: T1, 413 cases (28.21%); T2, 473 cases (31.35%); and T3, 584 cases (39.89%) (*P* < 0.001). During follow-up, a higher AIP-FI was significantly associated with a progressively increased cumulative risk of arthritis (Log-rank *P* < 0.001), as shown in Fig. 2. Table 2 demonstrates the relationship between AIP-FI and arthritis risk. After adjusting for all covariates, each 1-unit increase in AIP-FI was associated with a 3% increase in arthritis risk (HR = 1.03; 95% CI: 1.02 to 1.04). When AIP-FI was analyzed by tertiles, participants in the highest tertile (T3) had a 55% higher risk compared to those in the lowest tertile (T1) (HR = 1.55; 95% CI: 1.35 to 1.78). The risk for those in the middle tertile (T2) was 17% higher than that in T1 (HR = 1.17; 95% CI: 1.02 to 1.33). As illustrated in Fig. 3, the Restricted Cubic Spline (RCS) curve revealed a nonlinear association between AIP-FI and arthritis risk. Notably, after adjusting for all covariates, a higher AIP-FI was consistently associated with an increased risk of arthritis (P for overall < 0.001, P for nonlinear < 0.001).

**Table 2:** Association between AIP-FI and arthritis risk in the COX regression model.

Variables	Model1		Model2		Model3	
	HR (95%CI)	*P*	HR (95%CI)	*P*	HR (95%CI)	*P*
AIP-FI	1.03 (1.02 ~ 1.04)	< 0.001	1.03 (1.02 ~ 1.03)	< 0.001	1.03 (1.02 ~ 1.04)	< 0.001
AIP-FI Tertile						
T1	1.00 (Reference)		1.00 (Reference)		1.00 (Reference)	
T2	1.14 (1.00 ~ 1.30)	0.051	1.15 (1.01 ~ 1.31)	0.043	1.17 (1.02 ~ 1.33)	0.024
T3	1.50 (1.32 ~ 1.70)	< 0.001	1.46 (1.29 ~ 1.66)	< 0.001	1.55 (1.35 ~ 1.78)	< 0.001

HR: Hazard Ratio, CI: Confidence Interval

Model1: Crude.

Model2: Adjust: Education, gender, place of residence, marital status, age.

Model3: Adjust: Diabetes, heart disease, stroke, dyslipidemia, BMI, hypertension, education, smoking, alcohol consumption, gender, place of residence, marital status, age.

### Subgroup analysis

**Fig. 4:**
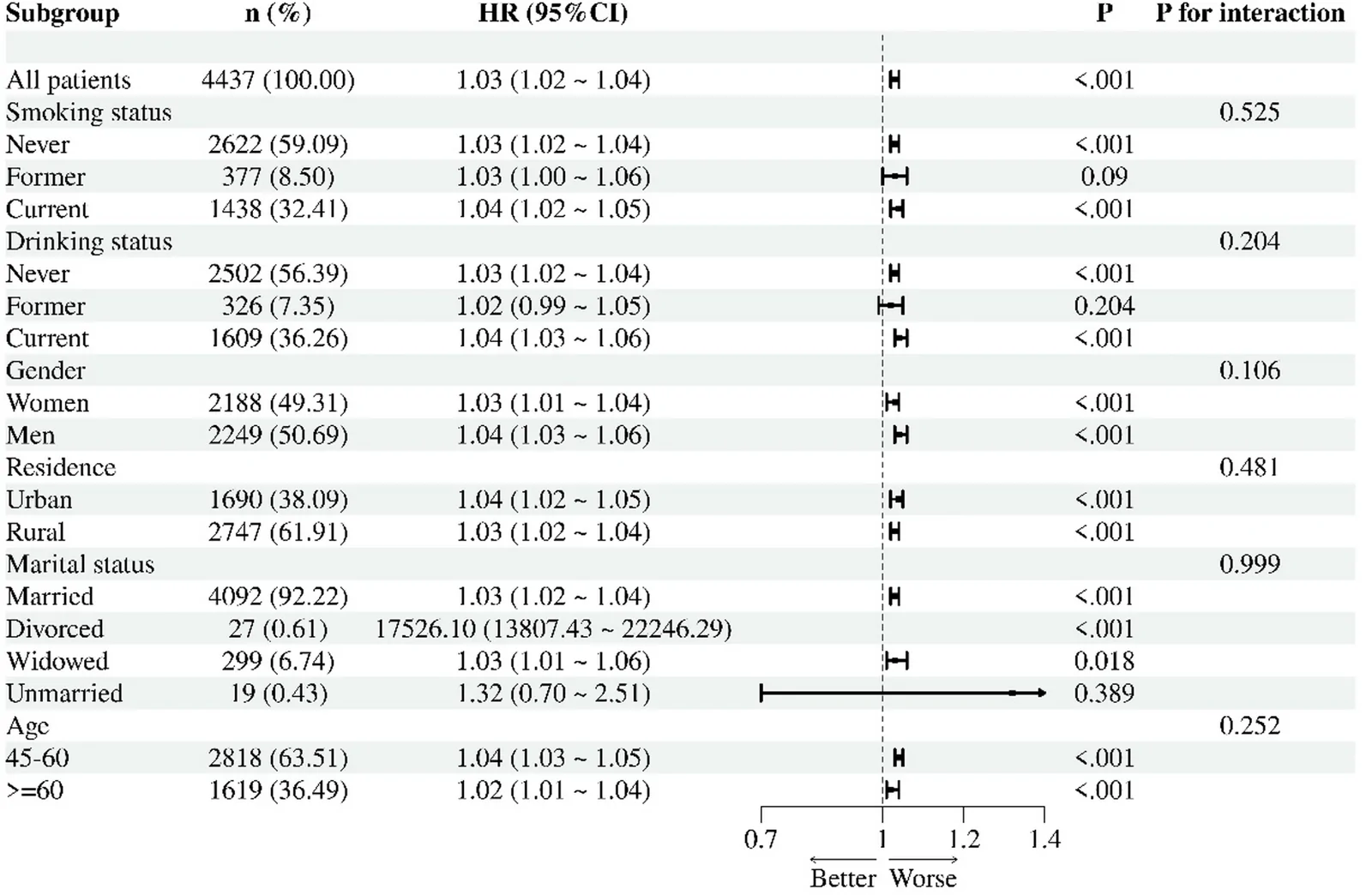

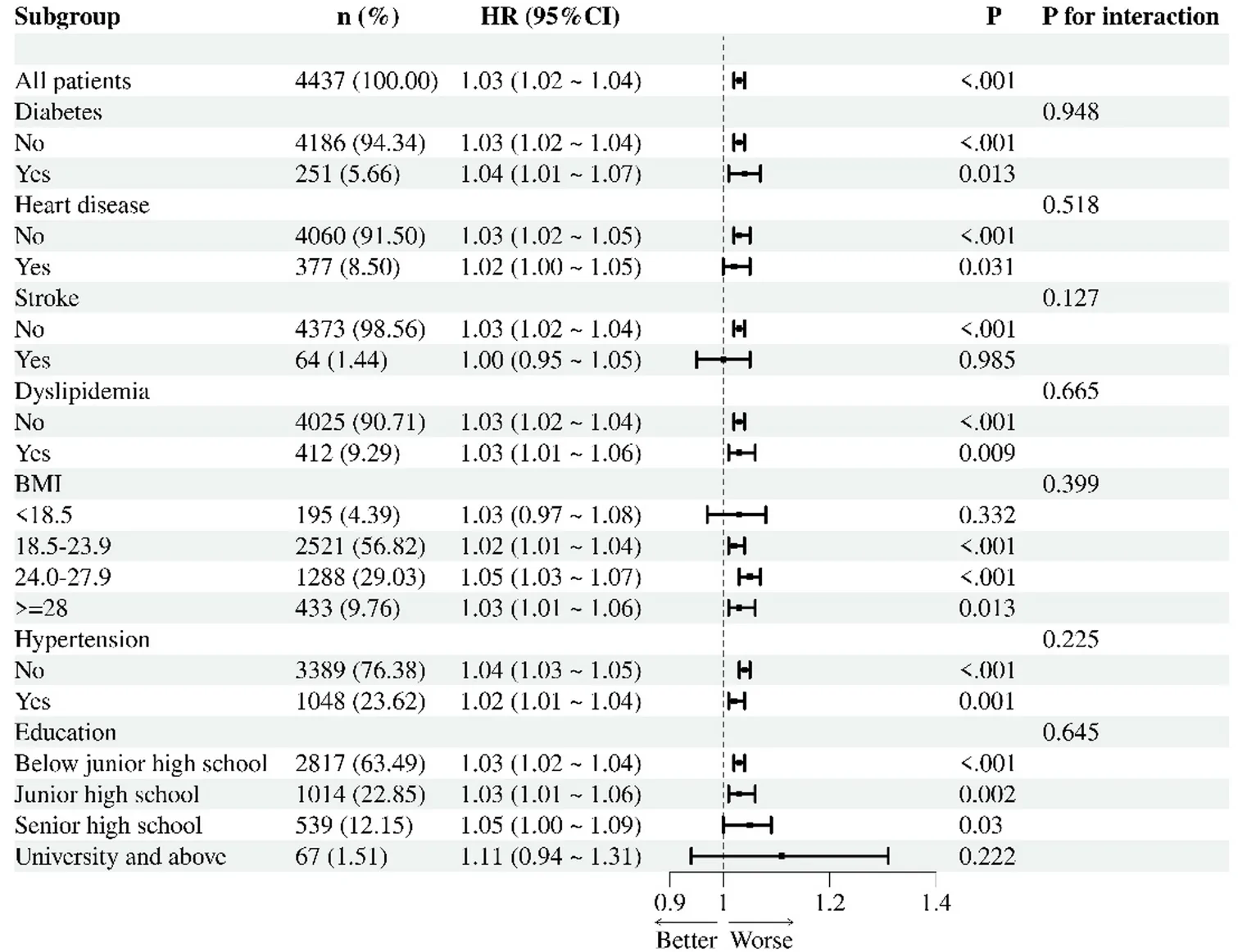
(**A**,**B**) Subgroup analysis.

Subgroup analyses, as shown in Fig. 4A and B, indicated that after adjusting for all covariates, the association between AIP-FI and arthritis risk was statistically significant in the overall population (HR = 1.03, 95% CI: 1.02–1.04, *P* < 0.001). When stratified by demographic characteristics, lifestyle factors, clinical comorbidities, and social factors, the direction of this association was consistent with the overall population in all subgroups (HR > 1). Moreover, all subgroup interaction P-values were > 0.05, suggesting no significant heterogeneity in the effect of AIP-FI on arthritis risk across different subgroups, indicating a consistent effect.

## Discussion

This study provides novel large-scale evidence on the relationship between the AIP-FI and the risk of arthritis among middle-aged and older adults in China. The level of AIP-FI was closely associated with an increased risk of arthritis, and this relationship remained significant even after comprehensive adjustment for covariates. The Kaplan-Meier curve further corroborated that a higher AIP-FI was associated with a greater risk of arthritis. Additionally, the restricted cubic spline curve revealed a significant nonlinear positive correlation between AIP-FI and arthritis risk. Subgroup analyses confirmed the consistency of the relationship between AIP-FI and arthritis risk across various population strata. In summary, this study suggests that AIP-FI was associated with arthritis risk and may warrant consideration as a potential indicator, pending further validation.

A prior study by Huo et al.^18^ demonstrated that a higher AIP-FI is significantly associated with an increased risk of cardiovascular diseases. Therefore, the AIP-FI may contribute to arthritis risk by affecting vascular function, which has been previously implicated in the pathophysiology of OA^25^. Research by Yoshimura et al.^26^ identified hypertension as a risk factor for the progression of knee OA (RR = 1.54), an association that persisted after adjusting for BMI and other metabolic factors. Ching et al.^27^ revealed that hypertension could disrupt joint homeostasis by exerting biophysical and biochemical effects on the synovium, subchondral bone, and chondrocytes, potentially acting as a pathogenic factor for OA. In the present study, the proportions of individuals with diabetes, hypertension, dyslipidemia, and BMI ≥ 28 kg/m² progressively increased from the T1 to T3 groups. Furthermore, in subgroup analyses, the hazard ratios (HR) for these covariates were > 1, suggesting that the AIP-FI might influence arthritis occurrence through these variables. Previous research has already established that these covariates can affect OA. Florent et al.^28^, in a study of 559 symptomatic knee OA patients aged over 50, found that type 2 diabetes is a predictor of joint space narrowing in male knee OA patients. Diabetes may interfere with the pathological process of arthritis through multiple mechanisms. Sustained hyperglycemia can induce non-enzymatic glycosylation of extracellular matrix proteins in cartilage, particularly affecting slowly renewing proteins like type II collagen^28,29^. This glycosylation alters the physical properties of proteins, increasing the stiffness of the cartilage collagen network and thereby reducing its resistance to mechanical stress^28,30^. Additionally, the association between diabetes and knee OA may involve reactive oxygen species (ROS) produced by OA chondrocytes, which increase under prolonged hyperglycemia and can induce degradation of cartilage matrix proteins^28,31^. Dyslipidemia may occlude blood vessels, leading to reduced energy supply to chondrocytes and contributing to arthritis formation. Meanwhile, a BMI ≥ 28 kg/m² falls into the obese category, and obesity is a recognized risk factor for arthritis development. The prevalence of OA is up to five times higher in obese individuals, and both its onset and severity are closely associated with weight status and duration of obesity^32^. Obesity is considered a chronic systemic pro-inflammatory state involving cytokines similar to those implicated in OA^33,34^. Previous studies indicate that mechanical loading, systemic inflammatory mediators derived from adipose tissue, and dysregulated metabolic mediators (such as adipokines, free fatty acids, advanced glycation end products, and glucotoxicity) can all increase the risk of obesity-induced OA^35,36^. Additionally, Xu et al.^37^ reported that AIP was negatively associated with trabecular bone score (TBS) after adjusting for covariates, suggesting a potential link between AIP per se and bone quality. Taken together, the effect of AIP-FI on arthritis may be mediated indirectly through vascular health, glucose metabolism, blood pressure, lipid profiles, and body weight, or alternatively through alterations in bone microarchitecture.

This study employed a composite measure—integrating the Atherogenic Index of Plasma (AIP) and the Frailty Index (FI)—to assess arthritis risk by combining metabolic and biological aging indicators. These findings suggest that a higher AIP-FI is associated with an increased risk of arthritis in middle-aged and older Chinese adults. However, as an observational study, this research cannot establish causality. The AIP-FI may serve as a potential indicator for identifying individuals at higher risk, but further prospective studies and clinical validation are needed before any practical application.

### Limitations

First, residual confounding may exist. Although we statistically adjusted for a wide range of known demographic, clinical, and lifestyle confounders in our analyses, some potential unmeasured confounding variables were not included in the models. Their absence may introduce a certain degree of bias into the current effect estimates. Second, the measurement of the outcome variable. In this study, the diagnosis of arthritis was based on patient self-report rather than objective confirmation via radiological imaging. While this method is practical in large-scale epidemiological studies, it may fail to identify asymptomatic early-stage cases and is susceptible to recall bias. This could lead to outcome misclassification, potentially diluting or distorting the true strength of the association. Third, sample representativeness and generalizability. Our analytical data were derived from the China CHARLS database, and the conclusions hold good internal validity within the context of the Chinese population. However, this population has specific characteristics in terms of genetic background, living environment, and healthcare accessibility. Therefore, the generalizability of our findings to other ethnic groups, geographical regions, or populations with different healthcare systems requires further validation in prospective, multi-center international cohorts. Fourth, the FI includes items related to mobility and activities of daily living, which may overlap with early symptoms of arthritis. Although individuals with self-reported arthritis were excluded at baseline, subclinical cases may still elevate FI scores through functional limitations. Nevertheless, the 10-year follow-up design and the objective nature of AIP may partially mitigate reverse causality. Future studies could further investigate this association by removing mobility-related FI items.

## Conclusion

In this cohort study, a higher AIP-FI was consistently associated with an increased risk of arthritis, with no significant heterogeneity observed across subgroups. This finding suggests that AIP-FI may be a potential indicator for identifying individuals at higher risk of arthritis. However, these results should be confirmed in independent cohorts before any practical application.

## Supplementary Information

Below is the link to the electronic supplementary material.

Supplementary Material 1

## Data Availability

The datasets generated and/or analysed during the current study are available in the China Health and Retirement Longitudinal Study repository, which can be accessed online at http://charls.pku.edu.cn. You can register as a CHARLS user to access all published data by following the necessary procedure.
